# The Effects of Iconicity and Conventionalization on Word Order Preferences

**DOI:** 10.1111/cogs.13203

**Published:** 2022-10-17

**Authors:** Yasamin Motamedi, Lucie Wolters, Marieke Schouwstra, Simon Kirby

**Affiliations:** ^1^ Centre for Language Evolution The University of Edinburgh; ^2^ Institute for Logic, Language and Computation University of Amsterdam

**Keywords:** Word order, Iconicity, Silent gesture, Conventionalization

## Abstract

Of the six possible orderings of the three main constituents of language (subject, verb, and object), two—SOV and SVO—are predominant cross‐linguistically. Previous research using the silent gesture paradigm in which hearing participants produce or respond to gestures without speech has shown that different factors such as reversibility, salience, and animacy can affect the preferences for different orders. Here, we test whether participants’ preferences for orders that are conditioned on the semantics of the event change depending on (i) the iconicity of individual gestural elements and (ii) the prior knowledge of a conventional lexicon. Our findings demonstrate the same preference for semantically conditioned word order found in previous studies, specifically that SOV and SVO are preferred differentially for different types of events. We do not find that iconicity of individual gestures affects participants’ ordering preferences; however, we do find that learning a lexicon leads to a stronger preference for SVO‐like orders overall. Finally, we compare our findings from English speakers, using an SVO‐dominant language, with data from speakers of an SOV‐dominant language, Turkish. We find that, while learning a lexicon leads to an increase in SVO preference for both sets of participants, this effect is mediated by language background and event type, suggesting that an interplay of factors together determines preferences for different ordering patterns. Taken together, our results support a view of word order as a gradient phenomenon responding to multiple biases.

## 1 Introduction

Languages differ in how they order the main constituents of subject (S), verb (V), and object (O), with some orders much more prevalent cross‐linguistically than others. In particular, just two orders—SVO and SOV—are the dominant ordering patterns for more than three quarters of the languages documented in the *World Atlas of Linguistic Structures* (Dryer, 2013) and are commonly attested dominant orders across different sign languages (Napoli & Sutton‐Spence, 2014). Furthermore, both SOV and SVO are predominantly produced in silent gesture experiments, in which hearing participants produce gestures without speech (Goldin‐Meadow, So, Ozyürek, & Mylander, 2008; Hall, Mayberry, & Ferreira, 2013; Meir et al., 2014; Schouwstra & de Swart, 2014). What can explain the preferences for certain word orders over others? Goldin‐Meadow et al. (2008) demonstrated that participants from different language backgrounds overwhelmingly produce gestures in SOV‐like sequences, which the authors suggest may reflect a fundamental preference to highlight concrete entities before relational concepts. Subsequent research using silent gestures paradigms has shown that ordering preferences can be modulated by different factors such as event semantics (Christensen, Fusaroli, & Tylén, 2016; Schouwstra & de Swart, 2014; Schouwstra, Swart, & Thompson, 2019), event reversibility (Gibson et al., 2013; Hall et al., 2013), the salience of event arguments (Kirton, Kirby, Smith, Culbertson, & Schouwstra, 2021), and animacy (Meir et al., 2014).

**Fig. 1:**
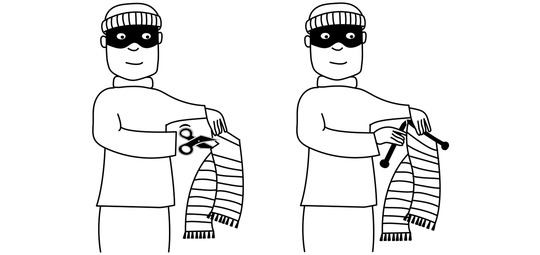
Example of events used in the experiments. (Left) An extensional event, depicting a burglar cutting a scarf. (Right) An intensional event, depicting a burglar knitting a scarf.

In particular, across several studies, Schouwstra and colleagues (Schouwstra & de Swart, 2014; Schouwstra et al., 2020, 2019) have demonstrated differential preferences for SOV and SVO‐like orders^1^ for two semantically distinct event types—extensional events and intensional events. Extensional events such as that depicted in the left‐hand panel of Fig. 1 (burglar‐cuts‐scarf) are those where the subject and object must be copresent for the event itself to take place. In contrast, for intensional events such as that depicted in the right‐hand panel of Fig. 1 (burglar‐knits‐scarf), the existence of the object is inherently tied to the event itself. This can be most clearly seen in the case of creation events such as *knit*, *bake*, and *draw*. In production and comprehension studies, as well as forced‐choice selection tasks, participants have expressed preferences for gestures ordered in SOV‐like sequences for extensional events and in SVO‐like sequences for intensional events (Schouwstra & de Swart, 2014; Schouwstra et al., 2020, 2019), reflecting a natural ordering preference conditioned on event semantics. However, few natural languages report such semantically conditioned ordering preferences, instead favoring systematic ordering patterns, where the same word order is used regardless of event semantics. More recently, evidence from two sign languages has demonstrated that such a distinction can emerge and persist in natural languages; research on both Brazilian Sign Language (Libras) and Nicaraguan Sign Language (NSL) has shown signers producing semantically conditioned ordering patterns similar to those found in laboratory experiments (Flaherty, Schouwstra, & Goldin‐Meadow, 2018; Napoli, Spence, & Quadros, 2017).

One possible explanation for the semantically conditioned preference relates to iconicity, such that the two orders better represent how the two types of events are enacted and perceived in the real world (Christensen et al., 2016). Extensional events, which require both subject and object to be copresent *before* the action occurs, are better represented with SOV‐like order, while intensional events, which typically require the action to occur for the object to exist,^2^ are better represented with SVO‐like order. In both cases, a bias for animate and, in particular, human entities lead to a preference for S to precede O (Meir et al., 2014; Prat‐Sala & Branigan, 2000; Van Nice & Dietrich, 2003; Vihman et al., 2018). This structural iconicity, sometimes referred to as *diagrammatic* iconicity (Meir & Tkachman, 2018), in which grammatical structures can represent the structure of real‐world events, is attested in both spoken and signed languages (Dingemanse, 2011; Hamano, 1998; Jakobson, 1965; Napoli & Sutton‐Spence, 2014; P. M. Perniss, 2007; Slonimska, Özyürek, & Capirci, 2020; Strickland et al., 2015; Wilbur, 2004) as well as experimental research (Christensen et al., 2016; Strickland et al., 2015). Structural iconicity can manifest independently of lexical iconicity (where individual signs are themselves iconic).

If iconicity does govern these preferences, we might expect that communication grounded in iconic *forms* (i.e., lexical iconicity) may prime participants (and possibly language users) to prefer iconic *structures*, consistent with the iconic mappings of the constituent parts. This is consistent with recent work showing that expectations for iconicity can themselves be learned (Sato, Schouwstra & Kirby, 2020 ). Moreover, while both iconic and arbitrary signals evolve over time through conventionalization processes (Caldwell & Smith, 2012; Fay, Garrod, Roberts, & Swoboda, 2013, 2010; Garrod, Fay, Lee, Oberlander, & MacLeod, 2007; Motamedi, Schouwstra, Smith, Culbertson, & Kirby, 2019; Theisen, Oberlander, & Kirby, 2010), arbitrary signals in particular must be learnt as part of a lexicon to be understood, whereas iconic signals may be understood more easily by a naive interlocutor, based on shared experience and world knowledge (Fay, Arbib, & Garrod, 2013; Klima & Bellugi, 1979; Lockwood, Dingemanse, & Hagoort, 2016; Macuch Silva, Holler, Ozyurek, & Roberts, 2020; Perlman, Dale, & Lupyan, 2015; Sulik, 2018). Previous silent gesture studies have shown that the presence of existing conventions, in the form of a known or learnt lexicon, can lead to a shift in ordering preferences for extensional events towards a strong SVO preference, across different language backgrounds (Hall, Ferreira, & Mayberry, 2014; Langus & Nespor, 2010; Marno et al., 2015). Therefore, while we might expect that iconic versus arbitrary mappings themselves affect preferences for different constituent orders, the necessity of learning a conventionalized lexicon may further govern the orders that language users prefer.

In summary, the evidence from both natural languages and experimental research suggests that how we order the basic constituents of language are influenced by the interplay of different factors related to event representations, linguistic context, and the social‐communicative function of language. Here, we investigate how preferences for semantically conditioned orders, as found by Schouwstra and colleagues (Schouwstra & de Swart, 2014; Schouwstra et al., 2020, 2019), are affected by iconicity, conventionalization, and prior linguistic experience. Across the set of experiments we report, we use an online forced‐choice paradigm to test participants’ ordering preferences for extensional and intensional events. In Experiment 1, we ask whether the previously reported preferences for English speakers, such that they prefer SVO‐like orders for intensional events and SOV‐like orders for extensional events, differ depending on whether they are shown sequences of iconic gestures compared to sequences of arbitrary gestures. We follow a procedure similar to that used by Marno et al. (2015)—participants are first taught a lexicon of the individual gestures for constituent parts before being asked to select a preferred gesture sequence for the full event. Teaching the lexicon before the sequence selection task ensures that participants understand the individual constituent parts and thus their responses to the gesture sequences reflect their preferences for different constituent orders. However, this also allows us to ask whether conventionality, in the form of a known lexicon, leads to an overall preference for SVO‐like orders, as Marno et al. (2015) found across speakers from different language backgrounds. In Experiment 2, we ask what the source of the SVO preference is by seeing how the hypothesized shift in preference differs in speakers from a language with a different basic order, comparing findings from Experiment 1 with data from Turkish speakers, an SOV‐dominant language.

## 2 Experiment 1: The effect of iconicity and conventionality on English speakers’ ordering preferences

We ran an experiment in which participants were taught individual gestures for the component parts of an event in a lexicon training stage and then asked to select a gesture sequence describing the full event in a forced‐choice task and a rating task. We compare data from this study with a previously run study (described in detail by Motamedi, Wolters, Naegeli, Kirby, & Schouwstra, 2022), in which participants completed just the forced‐choice task and rating task for iconic gestures, without being taught a gestural lexicon.

**Fig. 2:**
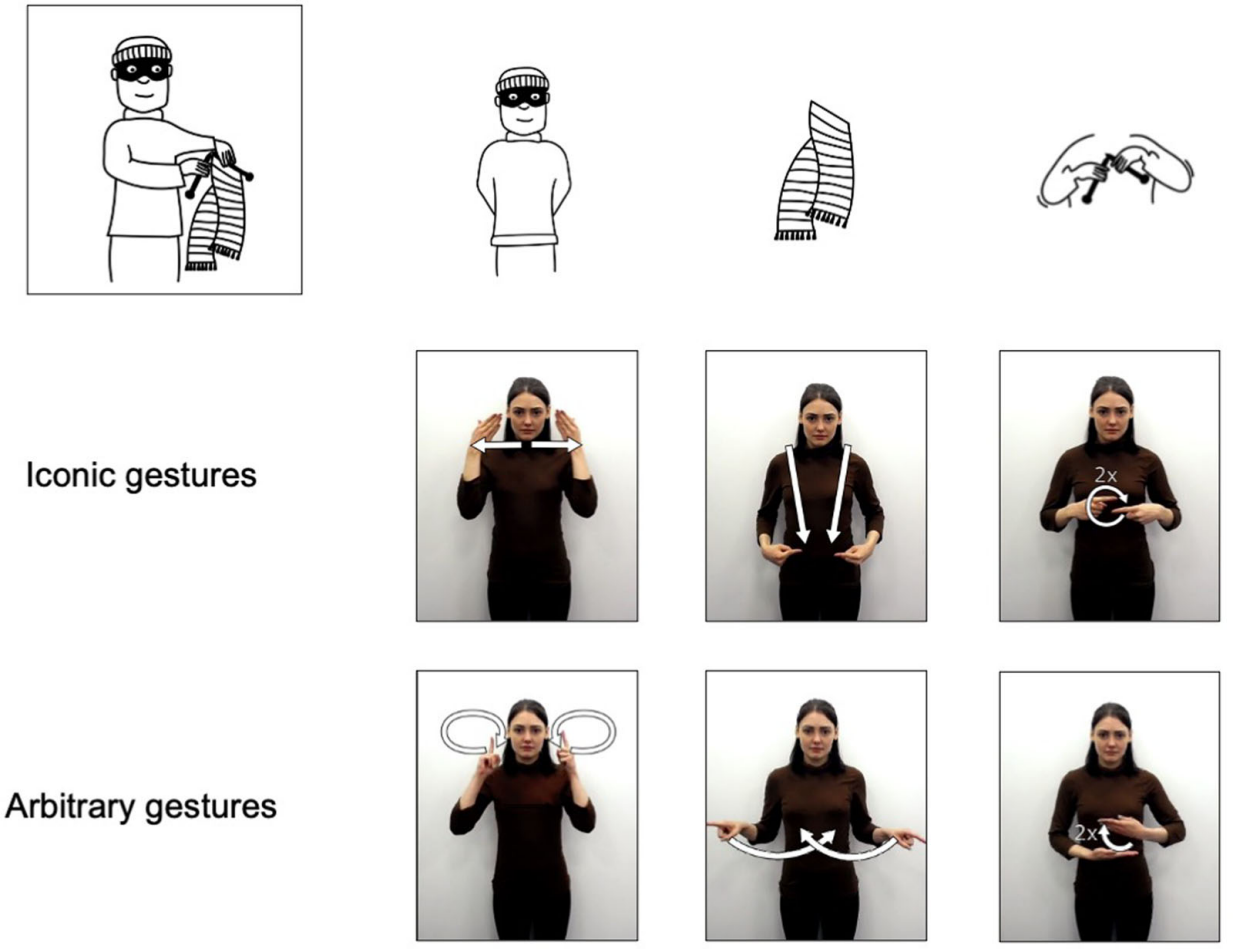
Examples of iconic and arbitrary gestures used for the event burglar‐knits‐scarf. Each constituent part of the event has its own iconic and arbitrary gesture. Arbitrary gestures were designed to match iconic gestures for complexity.

We use the same set of events described by Motamedi et al. (2022), comprising four actor–object scenarios which appear as part of an intensional or an extensional event (see Table 1), and which were presented as line drawings. Each event can be communicated with sequences of gestures describing each component part (actor, object, action). We first devised a set of iconic gestures for each event's component parts, and then devised arbitrary gestures that did not bear resemblance to the component parts of the event, but which matched corresponding iconic gestures based on handedness, location, and handshape complexity. An example of both iconic and arbitrary gesture sequences for the same event is shown in Fig. 2.

**Table 1:** Constituent parts of events used in the experiment

Actor	Object	Extensional Event	Intensional Event
nun	ukelele	throw	think
burglar	scarf	cut	knit
chef	pram	push	dream
gnome	banana	eat	paint

### 2.1 Gesture norming

To ensure that the gestures we devised were perceived as intended, we ran a gesture norming task in which 20 naive participants were asked to rate individual constituent gestures for iconicity. We have a total of 32 unique gestures, with one iconic and one arbitrary gesture for all items in Table 1. Participants rated all 32 unique gestures for iconicity on a scale of 0–100, with the right–left endpoints of the scale randomized for each participant to represent either the iconic or arbitrary extreme. To explain iconicity and arbitrariness, participants were first shown videos of two signs from British Sign Language, one highly iconic (DRINK) and one arbitrary sign (BROTHER). They were instructed that they would be shown signs from an artificial sign language and that they should indicate how iconic or arbitrary the sign is by moving the slider.

Data from the study were rescaled such that, for all participants, zero represented the most arbitrary value and 100 the most iconic. We ran a mixed effects linear model with R (R Core Team, 2013) and lme4 (Bates, Bolker, Machler, & Walker, 2015), analyzing the effect of intended iconicity (deviation coded as −0.5 = intended arbitrary, 0.5 = intended iconic) on participants’ ratings, including by‐participant and by‐item random intercepts. Our model indicated improved fit over the null model (χ^2^ = 44.31, *p* < .001), and model results suggested that gestures intended to be iconic were rated as significantly more iconic than gestures intended to be arbitrary (β = 22.02, *SE* = 2.27, *t* = 9.72, *p* < .001).^3^

### 2.2 Main experiment design and procedure^4^

A total of 330 participants took part in the main study, an online task in which participants were taught individual gestures for the component parts of an event in a lexicon training stage and then asked to select a gesture sequence describing the full event in a forced‐choice task and a slider rating task. Participants were recruited from the crowdsourcing platform Prolific and were pre‐screened to allow English‐speaking participants to take part. The experiment took 3 min to complete, and participants were compensated £1.10 for their time. We received ethical approval for all studies described here and throughout from the Philosophy, Psychology and Language Sciences (PPLS) ethical review board at the University of Edinburgh. Informed consent was obtained from all participants prior to data collection.

**Fig. 3:**
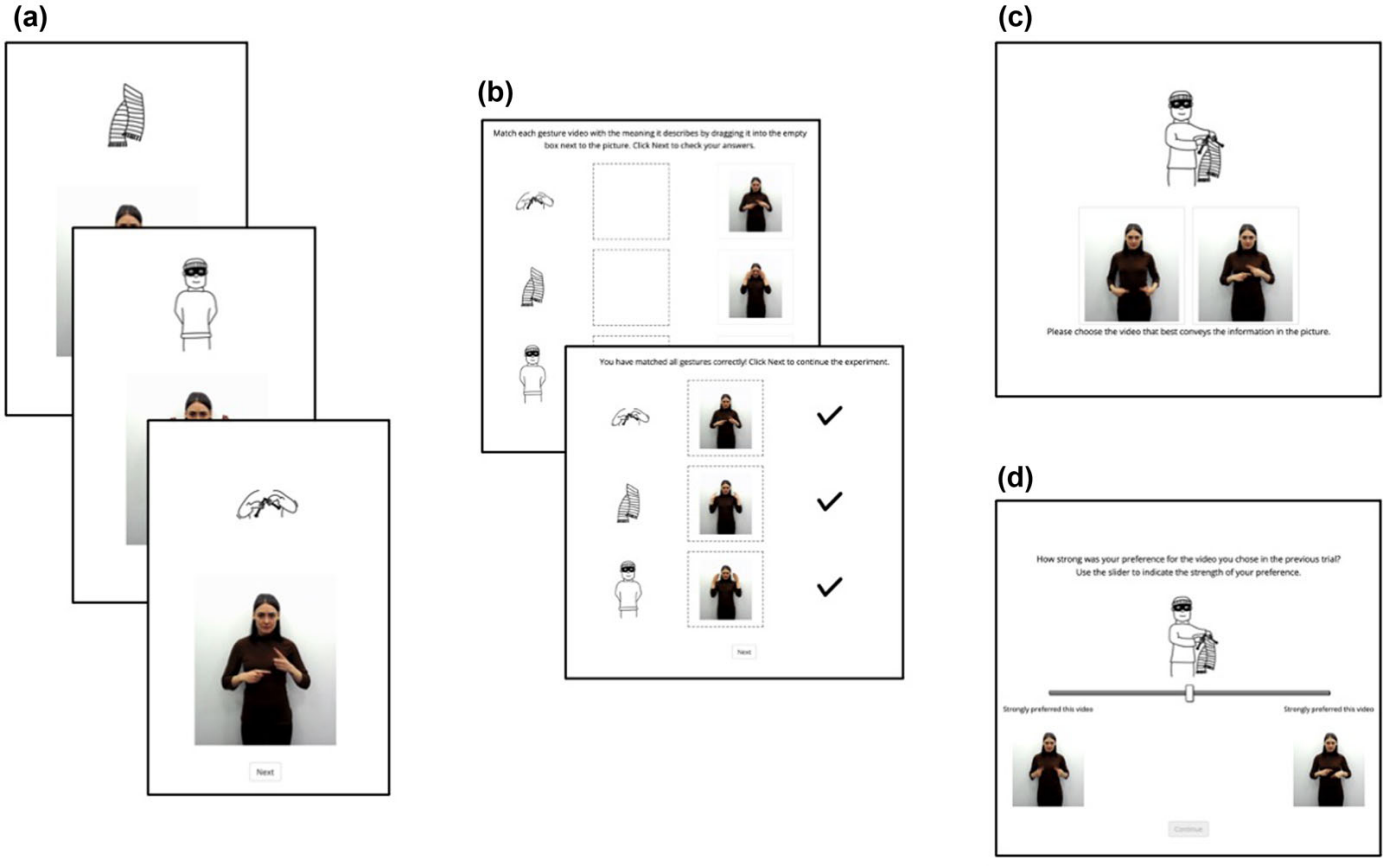
Experimental procedure. Participants were first trained on individual gestures for the constituent parts of events (a), before completing a matching task to test their knowledge (b). In the main part of the experiment, participants completed a selection task (c), where they had to choose from one of two ordered gesture sequences, and a slider task (d), where they had to indicate the strength of their preference made in the selection task.

Participants completed the task in relation to a single event, which could either be extensional or intensional, and in response to gestures that were either iconic or arbitrary, with random assignment to event type and condition. In the first part of the task, the *lexicon training stage*, participants were first trained on individual gestures describing the component parts of each event. We included lexicon training to ensure that participants’ selections in the main task reflect their ordering preferences and not a misunderstanding of the meaning of each gesture. This was essential in the arbitrary condition since by design participants had no other way to determine which part of the gesture sequence corresponded to which part of the meaning. Participants were shown each component part of the event separately as a line drawing, with a looping gif of its corresponding gesture (Fig. 3A). Participants could watch the gesture as many times as they liked, but they had to watch it at least twice all the way through before they could continue. To test whether they had learnt the gestures, participants had to match the component part to its corresponding gesture and could not progress to the main task until they had correctly matched all three components (Fig. 3B). As the main part of the experiment focuses on orders similar to SVO and SOV ordering patterns, the order of presentation for component parts in the lexicon training stage was randomly selected for each participant from the remaining possible basic ordering patterns (OSV, OVS, VSO, VOS) to minimize the risk of unwanted priming of gesture sequence order.

In the main part of the experiment, participants completed a *selection task* and a *slider task*. In the selection task, participants were shown the line drawing of the full event, with two gesture videos shown underneath, communicating the full event (see Fig. 3C). All full‐event videos were approximately 4.5 s in length. The gestures shown in each video were identical except for the order in which the gestures for component parts were produced—one video showed a gesture sequence of SOV‐like order, and the other a sequence of SVO‐like order. Both videos played on a continuous loop, with videos synchronized such that the point of segmentation of actor, object, and action was the same in both videos. Participants were asked to select which of the two videos best conveyed the event shown in the image, by clicking on either video. Following the selection task, participants completed a slider task in which they were shown a slider as well as the event image and both videos again (see Fig. 3D). They were asked to move the slider to indicate the strength of preference for the video chosen in the selection task. The left‐right position of each ordering variant was randomized per‐participant but remained consistent across both the selection and slider tasks.

Finally, we asked participants to complete the matching part of the lexicon training stage again to check their understanding of the mapping between each gesture and its corresponding component part.

Following data collection, we excluded participants according to three criteria. First, we excluded participants who responded to the selection task in under 9 s, too quickly to see the different orders in the two videos. Second, we exclude participants whose preference indicated in the slider task was different to that indicated in the selection task (e.g., they select the SOV‐like video, but indicate preference in favor of the SVO‐like video). Finally, we excluded participants who failed to correctly match component parts to their corresponding gestures in the final matching task. After exclusions, we had a total of 254 participants, distributed as follows across conditions: extensional‐iconic = 64, extensional‐arbitrary = 67, intensional‐iconic = 59, intensional‐arbitrary = 68.

## 3 Results

**Fig. 4:**
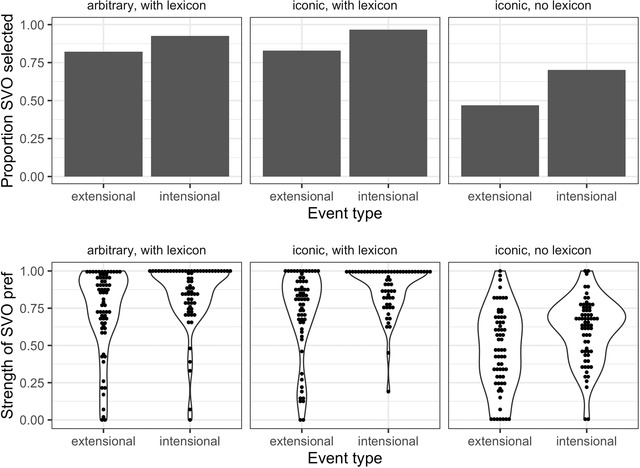
Results from Experiment 1, showing the proportion of the SVO‐variant selected in the selection task (top row) and the strength of preferences expressed for the SVO‐variant in the slider task (bottom row). Participants prefer the SVO‐variant more often for intensional events compared to extensional ones, but the overall preference for the SVO‐variant is higher when participants learn the gestural lexicon before making their selection.

Our findings from both the selection and slider tasks are shown in the left‐hand and middle panels of Fig. 4. We analyzed data from the selection task using a logistic regression model, predicting the preference for the SVO‐like variant over the SOV‐like variant, including event type (extensional/intensional) and iconicity (iconic/arbitrary) as deviation‐coded predictors, along with their interaction. Model comparison indicated that the model including the effect of event type improved the fit over the null model, but additional predictors did not (χ^2^ = –9.56, *p* = .002), suggesting that the iconicity of the gestures does not significantly affect participants’ ordering preferences. Inspection of the model revealed an overall preference for the SVO‐like variant (β = 2.19, *SE* = 0.23, *z* = 9.72, *p* < .001) and an increased SVO preference for intensional events compared to extensional events (β = 1.29, *SE* = 0.45, *z* = 2.87, *p* = .004).

Data from the slider task were transformed such that the values represented the strength of preference for the SVO‐variant and analyzed using a linear regression model predicting the transformed scores from event type, iconicity, and their interaction, deviation coded as above. The model analysis showed similar findings to the selection task; a model including the event‐type predictor improved fit over the null model (*p* = .002), but further predictors did not significantly improve fit. The model indicated a higher SVO preference for intensional compared to extensional events (β = 0.14, *SE* = 0.03, *t* = 4.55, *p* < .001).

### 3.1 Comparison with no‐lexicon experiment

Overall, our findings are consistent with previous silent gesture production and comprehension tasks, in which participants indicate stronger preferences for SVO‐like gestures for intensional events and stronger preferences for SOV‐like gestures for extensional events. In the present study, whether the gestures are iconic or arbitrary has little effect on participants’ ordering preferences, failing to support our hypothesis that structural iconicity would be modulated by the iconicity of the gestures themselves. However, in order to compare participants’ preferences for iconic and arbitrary gestures on an even footing, we needed to teach participants in the present study the gestural lexicon before showing them the ordered sequences even for the iconic gestures, which could also have an effect on their overall preferences. Here, we compare participants’ responses in the present study, where they respond after learning a lexicon, to participants’ responses from a previous study in which they responded to sequences of iconic gestures without first learning a lexicon. Note that for the iconic condition, these two experiments are identical except for the first stage, where the meaning of the individual iconic gestures is shown to participants.

We use data collected from a study reported by Motamedi et al. (2022)^5^ from 160 participants recruited through Prolific for an online task. The experiment they completed was identical to the iconic condition of the experiment detailed above, except in the following details: (i) participants did not complete a lexicon training stage, but after the instructions and consent form proceeded straight to the selection and slider tasks and (ii) participants did not complete a final lexicon matching test. Participants were excluded as before based on whether they responded too quickly in the selection task and whether the preference expressed in the slider task did not match their choice in the selection task, leaving a total of 141 participants (*N*_extensional_ = 70, *N*_intensional_ = 71).

We compare data from across the three datasets (illustrated in Fig. 4)—iconic gestures without lexicon training, iconic gestures with lexicon training, and arbitrary gestures with lexicon training—using mixed effects modeling.

For the selection task, we used a logistic regression model including deviation‐coded fixed effects of event type, iconicity and whether or not participants completed a lexicon training stage, as well as interactions between event type and iconicity and event type and lexicon training. We also included a by‐item random intercept. The model including fixed effects of event type and lexicon training demonstrated improved fit over a model without lexicon training (χ^2^ = 48.73, *p* < .001), but additional fixed effects, including the effect of iconicity and the interaction terms, did not further improve model fit. Inspection of the model revealed a significant intercept, suggesting an overall preference for the SVO variant (β = 1.30, *SE* = 0.22, *z* = 5.86, *p* < .001). We also found a significant effect of event type, with intensional events eliciting a higher proportion of SVO selections (β = 1.15, *SE* = 0.44, *z* = 2.59, *p* = .01), and a significant effect of lexicon training, such that the inclusion of the lexicon training stage led to a higher proportion of SVO selections overall (β = 1.90, *SE* = 0.29, *z* = 6.62, *p* < .001).

We use an identical model structure for the slider task, predicting participants’ slider scores using a linear model. Aligning with the findings from the selection task, a model including fixed effects of event type and lexicon training improved fit over a reduced model (χ^2^ = 94.83, *p* < .001), and additional fixed effects did not further improve fit. The model results demonstrated an increased preference for the SVO variant both for intensional events (β = 0.13, *SE* = 0.04, *t* = 3.27, *p* = .02) and when participants completed a lexicon training stage (β = 0.27, *SE* = 0.03, *t* = 10.43, *p* < .001).

### 3.2 Interim summary

We investigated whether the iconicity of gestural signals affects participants’ ordering preferences for sequences communicating extensional and intensional events. While we replicate previous findings showing that ordering preference is conditioned on the type of event participants see, with participants showing a higher preference for SVO‐like order for intensional versus extensional events, contrary to expectations, we do not find that the iconicity of gestures has a significant effect on participants’ responses. Moreover, to test the effect of iconic and arbitrary gestures equally, we initially taught participants a lexicon of individual gestures before showing them the gesture sequences in different orders. Despite this apparently minor change to the design, when compared with a study in which participants expressed their ordering preferences for iconic gesture sequences without first learning a lexicon, we found that the lexicon training leads to an overall increase in preference for the SVO‐like sequence, in line with previous findings by Marno et al. (2015) for extensional events. However, Experiment 1 sampled English‐speaking participants, who use an SVO‐dominant language. We wondered if the effect of native language might explain the increased preference in SVO overall. In Experiment 2, we compare data from Experiment 1 with data collected from speakers of Turkish, an SOV‐dominant language.

## 4 Experiment 2: Comparing the effect of conventionality on ordering preferences for English and Turkish speakers

### 4.1 Participants and methods

We ran two studies in which Turkish‐speaking participants were shown sequences of iconic gestures in SVO‐ and SOV‐like orders and asked to indicate which order they preferred for both extensional and intensional events.^6^ In one study, participants only complete the selection and slider tasks; in the other study, participants also complete the lexicon training stage and the final lexicon matching task, as in Experiment 1. Given constraints on the number of participants we were able to recruit, we focused here on iconic gestures with or without a lexicon, as Experiment 1 did not demonstrate a reliable difference in ordering preferences for iconic and arbitrary gestures. All materials and procedure are identical to that described for Experiment 1, with the exception that all text was translated into Turkish by a fluent, first‐language Turkish speaker.

We collected data from 168 participants, who were randomly assigned to either the study with lexicon learning or without lexicon learning. Using the same exclusion criteria as Experiment 1, we excluded 28 participants, leaving a total sample of 140 participants, distributed as follows: extensional‐lexicon = 36, extensional‐nolexicon = 33, intesional‐lexicon = 35, intensional‐nolexicon = 36.

### 4.2 Results

**Fig. 5:**
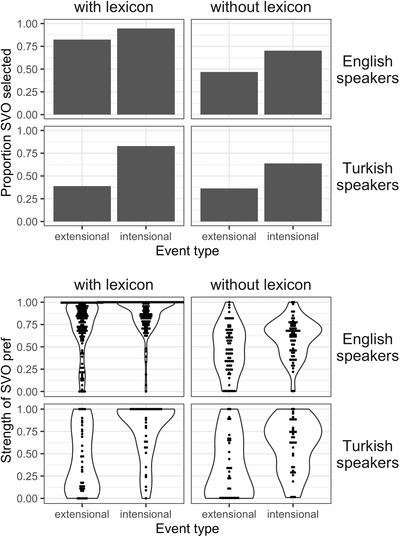
Results from Experiments 1 and 2 showing, for both English and Turkish speakers, the proportion of SVO selections made in the selection task (top panel) and the strength of preference for the SVO‐variant in the slider task (bottom panel). Both groups show a higher overall SVO preference when participants first learn a lexicon, but this shift is mediated by event type and language background.

We compare the data collected here from Turkish speakers with corresponding data from Experiment 1 collected from English speakers (iconic gestures, with and without lexicon learning). Results from the selection and slider tasks are illustrated in Fig. 5.

We analyzed data from across the studies using mixed effects regression models. For the selection task, we used a logistic regression, including event type, lexicon training, and language as deviation‐coded fixed effects, along with all interaction terms, as well as a by‐item random intercept. Model comparison indicated that the model without the three‐way interaction (but including all simple interactions) improved fit over a reduced model with only main effects (χ^2^ = 11.57, *p* = .009)—including the three‐way interaction did not further improve model fit. The model demonstrated an overall preference for the SVO variant (intercept: β = 0.79, *SE* = 0.20, *z* = 3.90, *p* < .001). Overall, we find a main effect of event type (β = 1.40, *SE* = 0.40, *z* = 3.48, *p* < .001), such that participants from different language backgrounds show increased preference for the SVO variant when shown intensional events compared to extensional events, as well as a main effect of lexicon learning (β = 1.29, *SE* = 0.25, *z* = 5.26, *p* < .001), such that including lexicon learning increases the overall preference for the SVO variant. In addition, we find a main effect of language (β = –1.09, *SE* = 0.24, *z* = –4.57, *p* < .001), with Turkish speakers showing an overall lower preference for the SVO variant compared to English speakers. Finally, we find a significant interaction between language and lexicon training (β = –1.39, *SE* = 0.48, *z* = –2.91, *p* = .004); the effect of including lexicon training in increasing the SVO preference is smaller for Turkish speakers than for English speakers.

We analyze data from the slider task using an identical model structure in a linear regression model. As with the selection task, model comparison indicated that the model without the three‐way interaction (but including all simple interactions) improved model fit over the reduced model with only main effects (χ^2^ = 18.02, *p* < .001). Inspection of the model revealed similar main effects of event type (β = 0.21, *SE* = 0.05, *t* = 4.40, *p* = .003), lexicon training (β = 0.19, *SE* = 0.03, *t* = 7.28, *p* < .001) and language (β = –0.14, *SE* = 0.03, *t* = –5.15, *p* < 0.001), and, aligning with data from the selection task, a significant interaction between language and lexicon training (β = –0.15, *SE* = 0.05, *t* = –2.81, *p* = 0.005). We further find an interaction between event type and language (β = 0.16, *SE* = 0.05, *t* = 3.13, *p* = .002), such that the difference in the strength of preference between intensional and extensional events is larger for Turkish speakers compared to English speakers.

### 4.3 Interim summary

In Experiment 2, we compared results from English speakers, who use an SVO‐dominant language, with Turkish speakers, whose language has SOV as the dominant word order. We find that, across both types of speakers, participants prefer SVO‐like ordering for intensional events compared to extensional ones, but the preference for SVO‐like orders across both events increases when participants learn a lexicon. However, this increase is smaller for Turkish speakers, where the increase in SVO selections is more extreme for intensional events, where SVO is consistent with natural ordering preference. When asked about the strength of their preference for each order, Turkish participants show a larger difference between extensional and intensional events than do English‐speaking participants.

## 5 Discussion

Across the studies presented here, we have investigated how four different factors—event type, iconicity, conventionality, and language background—affect participants’ ordering preferences for different events. Overall, our findings pertaining to event type are consistent with previous studies, such that participants show an increased preference for SVO‐like orders for intensional events compared to extensional events (Schouwstra & de Swart, 2014; Schouwstra et al., 2020). This preference in itself has been previously explained as relating to structural iconicity, with different orders better representing real‐world events. Under this account (Christensen et al., 2016), extensional events require both subject and object to be physically present for the event to take place, and thus are better represented by SOV‐like orders. In contrast, intensional events usually require the event to take place for the object to exist and are iconically reflected in SVO‐like structures.

In addition, the semantically conditioned ordering preference we find here and in other studies is not yet widely reported in natural languages but has been thus far reported in two sign languages: Libras (Napoli et al., 2017), and NSL (Flaherty et al., 2018). Sign languages are highly visually iconic (Emmorey, 2014; P. Perniss, Thompson, & Vigliocco, 2010; Taub, 2001), and this iconicity influences event representation in comparison to spoken languages. Cross‐linguistically, different sign languages use simultaneous articulation and structural iconicity to represent properties of events (Emmorey, 2014; P. M. Perniss, 2007; Slonimska et al., 2020), and the lexical iconicity of individual signs can influence preferred ordering patterns (Napoli & Sutton‐Spence, 2014). We hypothesized that lexical iconicity in our study could therefore play a role in influencing ordering preferences for extensional versus intensional events, such that arbitrary gestures, in reducing the emphasis on iconic representation, would in turn– deemphasize the structurally iconic affordances of each event type.

However, our findings suggest that this is not the case. We could not confirm a statistical difference between learners of iconic versus arbitrary gestures, though participants still selected the SVO‐variant for intensional events more frequently than extensional events. Our finding that semantic conditioning is preferred, even when the lexical material is not iconic, is consistent with results from a previous experiment not involving silent gesture in which participants had to match pictures of events with written descriptions (Schouwstra, Naegeli, & Kirby, in preparation^2022^). Participants selected from different paratactic orders, where the constituent parts of events were described as a sequence of three short sentences. For example, the event *burglar‐knits‐scarf* would be represented in an SVO‐like paratactic construction as *There is a burglar/The burglar knits something/It is a scarf* and in an SOV‐like construction as *There is a burglar/There is a scarf/The burglar knits the scarf*. In this case, where the iconicity of the constituent parts cannot come into play, the authors report a similar preference for semantically conditioned orders. This suggests perhaps that the mechanisms that drive the kind of structural iconicity involved in word order are different from those involved in the iconicity of individual gestures.

In the context of our study, to compare iconic and arbitrary gestures on an equal footing, we introduced the lexicon learning stage, in which participants learnt the individual constituent gestures as conventional mappings before responding in the main task. By teaching participants the conventional lexicon first, we were able to focus our interpretation of results from the main task solely in terms of iconicity rather than, for example, whether or not participants understood what the constituent gestures referred to in each condition. We compared data in Experiment 1 with data from a previous experiment using iconic gestures without the lexicon learning stage, allowing us to test the effect that learning a lexicon has on ordering preferences. In this case, the inclusion of lexical training led to an increase in the overall preference for SVO order in both English speakers (who use an SVO‐dominant language) and Turkish speakers (who use an SOV‐dominant language). Previous studies have demonstrated similar effects, such that drawing on an existing conventional lexicon leads to stronger preferences for VO ordering patterns, even in contexts that would otherwise elicit strong SOV preferences (Langus & Nespor, 2010; Marno et al., 2015). Langus and Nespor (2010) suggest that this shift reflects the ordering preference of the computational system of grammar, under which SVO order is “syntactically preferred.” They suggest that, in contrast, SOV order characterizes improvised communication and reflects an interaction between the conceptual system and sensori‐motor experience.

While our results align with the findings of both Langus and Nespor (2010) and Marno et al. (2015), we are reluctant to subscribe to the view that a single word order is syntactically preferred, sitting in contrast to other orders that are said to reflect prelinguistic constraints on communication. First, it is not clear what is meant by “syntactically preferred,” other than that they are relatively common, frequently reported as the dominant order for creole languages, and that the diachronic tendency is for languages to change from SOV to SVO and not the other way around. However, such a view downplays the prevalence for SOV orders cross‐linguistically in spoken languages and ignores the dominance of verb‐final orders in signed languages. In particular, SOV is suggested to be grammatical in all documented sign languages (Napoli & Sutton‐Spence, 2014); in contrast to the SVO dominance found in spoken creole languages, different emerging sign languages instead are suggested to use SOV order most frequently (Ergin, Meir, Aran, Padden, & Jackendoff, 2018; Flaherty, 2014; Sandler, Meir, Padden, & Aronoff, 2005). While SVO ordering patterns are suggested to be easier to process for reversible events, as they separate the two potentially ambiguous entities of S and O, languages may use other tools in addition to order, such as case marking, to disambiguate constituents and ease processing demands (Bentz & Christiansen, 2013; Ferrer‐I‐Cancho, 2014).

We suggest that the distinction between SVO as a syntactically preferred order and SOV as characteristic of pre‐linguistic communication fails to capture the full picture of what is going on, in contrast to, for example, Ferrer‐i‐Cancho's (2017) account of the different factors that influence natural language ordering preferences. He suggests an account of word order preferences that emerge due to the interaction between different cognitive constraints, such as syntactic dependency minimization and surprisal minimization (Ferrer‐i‐Cancho, 2017; Ferrer‐i‐Cancho, Gómez‐Rodríguez, Esteban, & Alemany‐Puig, 2022). Previous silent gesture research has also suggested that a preference for SVO orders can be explained with domain‐general accounts, namely a bias to place subjects first regardless of the other constituents, and the robustness to noise of SVO order, particularly for reversible events where subject and object can be confused (Gibson et al., 2013; Hall et al., 2014). Moreover, evidence from both natural languages and lab‐based experiments has shown that ordering preferences are sensitive to a wide range of language internal and external factors, including event semantics and reversibility, as well as animacy, the surrounding linguistic context, and the social‐interactive context in which language is used (Christensen et al., 2016; Gibson et al., 2013; Kirton et al., 2021; Levshina et al., 2021; Meir et al., 2014; Napoli & Sutton‐Spence, 2014; Schouwstra & de Swart, 2014). Ordering patterns within and across languages will reflect this interplay of different factors.

We suggest that our findings attest to this. While we do find that learning a conventional lexicon leads to an overall increase in a preference for SVO‐like orders, this preference is mediated both by event type and by the language background of our participants. Importantly, the effect of event type found previously across several studies persists, suggesting that the preference for grammatical structures that reflect the natural structure of events interacts with the process of learning a set of conventionalized forms in a lexicon. Moreover, we find that speakers of an SOV‐dominant language (Turkish) do not respond to the lexicon learning task in the same way as speakers of an already SVO‐dominant language (English). Instead, the increase in the SVO preference with the conventional lexicon is stronger for intensional events, where it is consistent with the natural preference for semantically conditioned order, compared to extensional events, where it conflicts with the natural preference. Turkish speakers also show a larger difference between extensional and intensional events when asked about the *strength* of their preferences, though this does not lead to significant differences in the binary selections they make. Further study could investigate how more variable usage of different ordering patterns may reflect more variable preferences compared to a binary forced‐choice task, but we suggest that, taken together, our findings reflect the multiplicity of factors that affect word order preferences (Hall et al., 2014) and suggest not a separation of the conceptual‐linguistic system from “pre‐linguistic,” domain‐general systems (e.g., perceptual, sensori‐motor), but rather an interaction between them.

In conclusion, across the two studies we have presented here, we cannot confirm an influence of the iconicity of individual gestures on participants' preferences for semantically conditioned word orders (that reflect the natural structure of extensional and intensional events). We do find that the prior existence of a conventional lexicon does, in line with previous findings, lead to an overall increase in the preference for SVO‐like structures, but that this is itself mediated by the semantics of the event and by participants’ existing linguistic knowledge. This interplay of different factors in governing participants’ ordering preferences is consistent with the gradient and context‐dependent nature of ordering patterns in natural languages (Levshina et al., 2021; Napoli & Sutton‐Spence, 2014) and calls for a nuanced approach to the cognitive and contextual processes that influence language structure.

### Open Research Badges

This article has earned Open Data and Open Materials badges. Data are available at hdl.handle.net/10932/00‐057D‐0921‐30F0‐F201‐D and materials are available at https://osf.io/NREBJ/.

## Notes

1 We refer here to SOV‐ and SVO‐*like* orders for experimental contexts, as we do not assert that these systems necessarily have the distinct grammatical categories of “subject,” “verb,” “object.” Rather, we map constituent parts of these events onto the categories we expect them to correspond to in natural languages.

2 An exception to this definition of intensional events is the subclass of perception events, in which the action (seeing or hearing) is not required for the object to exist. In the current study, we did not include perception events. In general, object arguments of intensional verbs are understood more in terms of their meaning than of their reference and thus the objects of intensional verbs are potentially non‐existent or nonspecific (Forbes, 2020). This understanding poses challenges for conceptions of meaning in which reference plays a central role (Moltmann, 2020; Schwarz, 2020) and has led to arguments that a satisfactory definition of meaning in natural language may depend primarily on the analysis of intensional verbs (D'Ambrosio, 2019).

3 Significance values for coefficients from linear models here and throughout were obtained using the lmerTest package. Data and analysis scripts for the gesture norming and Experiment 1 can be found at https://osf.io/ny8u2/.

4 Experimental design and analysis plan were pre‐registered via the Open Science Framework at .

5 Data and analysis files relating to the comparison data can be found at https://osf.io/b9nm6/.

6 Pre‐registration of Experiment 2 can be found at https://osf.io/utafx. Data and analysis files can be found at https://osf.io/my9jq/.
